# Clinical application of optical coherence tomography angiography in diabetic macular edema

**DOI:** 10.4314/ahs.v23i2.56

**Published:** 2023-06

**Authors:** Meng Lv, Tuo Li, Yin Li

**Affiliations:** 1 Department of Ophthalmology, the Central Hospital of Enshi Autonomous Prefecture, Hubei University of Medicine, Shiyan 442000, Hubei Province, P.R.C; 2 Department of Ophthalmology, the Central Hospital of Enshi Autonomous Prefecture, Enshi Clinical College of Wuhan University, Enshi 445000, Hubei Province, P.R.C

**Keywords:** Diabetic macular edema, optical coherence tomography angiography, vascular endothelial growth factor, vessel density

## Abstract

Diabetic macular edema (DME) is characterized by a retinal thickening or hard exudation deposition in the fundus microvasculature, capillary leakage, increased vascular permeability, extracellular fluid accumulation in the fovea of a foveal disc. Optical coherence tomography angiography (OCTA) is a new item of fundus structure examination. OCTA is to reconstruct the retinal choroidal vascular structure from the continuous same cross-sectional views and blood flow signals obtained by optical scanning, thereby obtaining an image. It is very significant to evaluate, diagnose, treat and manage the disease.

## Introduction

Diabetes mellitus (DM) is a metabolic disease characterized by chronic hyperglycemia, and has many pathogenic factors 1. It is mainly caused by the absolute or relative deficiency of insulin secretion or the defect, which leads to the long-term metabolic disorder of carbohydrate, fat and protein. Therefore, it can cause multi system damage and lead to chronic progressive diseases of tissues and organs such as eyes, nerves, heart and blood vessels 2-3. Nowadays, due to the improvement of living standards, the increase of work pressure, the lack of daily activities, the gradual aging of the population and other factors, the prevalence of DM is increasing year by year 4. Among them, diabetic retinopathy (DR) is a progressive asymptomatic microvascular complication of DM, which can cause irreversible retinal damage, manifested by changes in the microcirculation of the eye, nerves and blood vessels 5. When DR involves the macula, capillary leakage, increased vascular permeability, extracellular fluid gradually accumulates in the macular fovea, retinal thickening or hard exudation deposition within the diameter of an optic disc will occur, which is called diabetic macular edema (DME) 6. Diabetic macular edema can be manifested as vision loss, accompanied by relative or absolute central scotomas, which is one of the main reasons for vision loss and even blindness in patients with DR 7. Optical coherence tomography angiography (OCTA) is a new inspection item of fundus structure. It has a unique and important working principle to obtaining an image 8. OCTA has important diagnostic value in DME, DR, retinal vein occlusion and other diseases. Here is a review of the clinical application of OCTA in diabetic macular edema.

## Pathogenesis of DME

Retinal Factors: Blood retinal barrier (BRB) is destroyed BRB is closely connected by retinal vascular endothelial cells, with a certain number of cell vesicles and fenestras, which keep the extracellular blood flow within a certain range and reduces the permeability exchange between cells 9-10. When the vascular permeability increases, extracellular fluid accumulates between the retinal nerve fiber layer and the retinal core layer, resulting in insufficient retinal blood perfusion, and the gradual development of the disease leads to macular edema 11. In addition, retinal vascular endothelial cells need the interaction between glial cells and pericytes to provide support and protection. Once glial cells or pericytes are lost or damaged, the permeability of endothelial cells will increase, resulting in the destruction and loss of retinal barrier function 12-13. The integrity of blood retinal structure and function is a necessary condition for maintaining the normal physiological function and activities of the retina. Various reasons lead to the destruction of barrier function, the accumulation of effusion between the retinal inner layer and the outer nuclear layer of the retina or retinal thickening 14, which all lead to macular edema.

### Vascular endothelial growth factor (VEGF and inflammatory factors increased

VEGF is a cytokine glycoprotein with dimer structure, which is mainly responsible for angiogenesis in physiological processes and angiogenesis in pathological processes 15. VEGF can regulate the proliferation and assembly of endothelial cells in normal physiological function, and exists in the whole angiogenesis cycle. However, when various pathological conditions occur due to diabetes or other diseases, VEGF levels are upregulated 16 of which may cause new angiogenesis, endothelial permeability, activation and release of various inflammatory mediators, fluid leakage into the vitreous body, and hemorrhage and fibrosis, which may destroy the internal environment of vascular stability 17-18.

### Metabolic Factors: Hypoxia and high glucose

When the blood glucose concentration is at a high level, the blood vessels have a relatively adaptive state to this high-level concentration. Under the stimulation of hyperglycemia, inflammatory factors are activated and released, such as tumor necrosis factor- α, IL-6, IL-8, VEGF and other inflammatory factors which will act further on the body. For example, the dysfunction of endothelial cells causes retinal microvascular and nerve, leading to the further aggravation of the disease 19. Breaking the balance between oxidation and antioxidation leads to oxidative stress. For example, the gradual accumulation of reactive oxygen species (ROS) can damage retinal blood vessels and retinal tissues 20. When the retina is hypoxic, the blood vessels in the macular region have their own regulation, resulting in the expansion of arterioles and the increase of internal pressure of venules and capillaries, so that some liquid in the blood vessels exudes and accumulates around the macula 21.

### Mechanical Factors: Vitreous traction

When there is fundus disease, the vitreous body will be concentrated or degraded. The appearance of vitreous membrane in the posterior pole will produce a forward traction effect on the retina. If this situation is not improved, the traction effect will always exist, and the fluid flowing into the tissue will continue to increase, resulting in macular edema 22-23.

### Observability and limitations of OCTA compared with traditional inspection methods

For fundus retinal diseases, the traditional examination method is fundus fluorescein angiography (FFA) 24. Research shows that although FFA can show various fundus conditions such as neovascularization, capillary leakage and micro angioma, due to its invasive operation, it needs to inject contrast agent during the examination process, which may have some side effects such as adverse reactions 25, which has certain limitations in clinical examination.

At present, OCTA has high resolution, fast, accurate and non-invasive observability. Vascular leakage and retinal hemorrhage will not have a great impact on the images obtained by blood flow scanning 26. Moreover, it can also carry out structured layered imaging of retinal vessels and choroidal vessels, qualitatively and quantitatively analyse the changes of blood vessels, reflect the changes of different colours according to the blood flow velocity, and analyse the possible abnormalities of blood vessels 27-28.

Although OCTA technology currently occupies the mainstream trend, and with further development, it can be used in more fields of research. However, FFA inspection cannot be completely replaced, because OCTA technology still has some limitations 29. The scanning range of OCTA is limited, and the examination of peripheral blood vessels is limited. In case of vascular leakage and retinal hemorrhage, the dynamic changes such as the speed of vascular filling cannot be reflected on the OCTA image 30-31. When the patient's coordination is poor and he can't focus on one point, artifacts will appear on the image results 32. In case of severe corneal edema, lens opacity and vitreous bloody effusion, it can be difficult to clearly image due to the turbidity of intraocular refractive medium 33.

## Clinical application of OCTA in DME

### Image features of normal retinal micro vessels displayed by OCTA

OCTA continuously scans the same cross section to monitor the blood movement, and processes the information such as blood flow density and blood flow changes, especially the characteristic changes of red blood cells in the blood flow 34. The research shows that in a specific area, the signal value of OCTA is positively correlated with the blood flow velocity. The stronger the signal value, the faster the flow rate 35. If the blood flow signal weakens or disappears when retinopathy occurs, a dark area will be displayed on the OCTA accordingly 36.

With the continuous development of OCTA technology, we can obtain fine images of retinal choroidal microvascular system, including its vascular perfusion defect, microvascular tumor, capillary remodelling, etc. In addition, OCTA also has the ability to analyse the blood flow in the macular area, monitor the blood flow density, and measure the area of the avascular area in the fovea of the macula.^37^.

### Macular vascular density (VD)

VD is a biomarker parameter in OCTA, which can be used to observe the movement and perfusion of retinal blood flow 38, it can reflect the severity of DME and play a certain role in disease diagnosis and monitoring. Vascular density can be divided into super capillary plexus (SCP), deep capillary plexus (DCP), radial capillary plexus around the optic disc (RPC) 39-40. Some studies have shown that the content of VD is closely related to the degree of retinopathy. The condition with low content of VD can develop to a more serious stage, thus increasing the incidence of DME 41. Foreign studies have found that the average level of VD in DME eyes is lower than that in normal eyes 42. Lee 43 further studied and analysed DCP and found that the content of VD in each division of DCP in the eyes of patients with DME decreased significantly, and this study also found that the content of VD in DCP is related to the treatment effect of diseases, which plays a guiding role in the diagnosis and prognosis of DME.

### Foveal avascular zone (FAZ)

FAZ is also used as a parameter of fundus retinal disease in OCTA. It is defined as the absence of any blood flow signal in the fovea of the macula, which can often be used as area, perimeter, blood flow density within 300µm width (fd-300), non circularity index (AI) 44-45. Studies have shown that compared with normal eyes, the FAZ area of DME affected eyes increases 46. FAZ area can also reflect the ischemia of DME affected eyes 47. The study by Attaallah found that 48 the FAZ area of SCP in DME eyes was larger than that in normal eyes, and the area was positively correlated with visual loss. The larger the FAZ area, the worse the vision.

### Evaluation of anti vascular endothelial growth factor (VEGF) therapy

In DME patients, various pathological conditions, such as diabetes or other diseases, can lead to VEGF up-regulated in the eye 49, which can promote angiogenesis, increase endothelial permeability, activate and release inflammatory mediators, and leak into the vitreous body, resulting in bleeding and fibrosis, which destroy the internal environment of vascular stability 50. In 1994, the study by Aiello found that 51 the level of intraocular VEGF in DR patients increased significantly, and the level of intraocular VEGF in DME and PDR patients reached 10 times that of normal people. Therefore, at present, the main treatment for DME is anti VEGF treatment by intraocular injection of drugs such as razumab or arbesip 52-53. The research shows that there is no significant change in VD in DME patients before and after anti VEGF treatment, indicating that this treatment will not aggravate macular ischemia, and is thus effective. Ghasemi 54 have also proved that the anti VEGF treatment will not aggravate the occlusion of ocular vessels in patients with DME through OCTA examination, and there is no significant increase in FAZ area. Through OCTA, the blood flow in macular region and other corresponding conditions can be observed in detail, so as to provide judgment and evaluation for the prognosis of DME patients undergoing anti VEGF treatment.

## Summary and Prospect

Nowadays, with its unique algorithm mode and scanning characteristics, OCTA can clearly image retinal choroidal vessels. It has been widely used in various fundus diseases, including DME, DR, retinal vein occlusion. The analysis of macular VD and FAZ and the characteristics of retinal blood flow can be used to reflect the diagnosis and prognosis of DME. However, there are still some deficiencies in OCTA as mentioned above. Therefore, it is still necessary to constantly explore and improve the index parameters and characteristics of OCTA and DME to further guide the evaluation, diagnosis, treatment and management of diseases.
